# Recent Evolution of the Intertidal Sand Ridge Lines of the Dongsha Shoal in the Modern Radial Sand Ridges, East China

**DOI:** 10.3390/ijerph18041573

**Published:** 2021-02-07

**Authors:** Binglin Liu, Haotian Wu, Zhenke Zhang, Guoen Wei, Yue Wang, Jie Zheng, Xuepeng Ji, Shengnan Jiang

**Affiliations:** 1 The School of Geographic and Oceanographic Sciences, Nanjing University, Nanjing 210023, China; DG1827018@smail.nju.edu.cn (B.L.); MG1927095@smail.nju.edu.cn (H.W.); dg1927034@smail.nju.edu.cn (G.W.); DG20270035@smail.nju.edu.cn (Y.W.); zq_jixuepeng@163.com (X.J.); DZ1627002@smail.nju.edu.cn (S.J.); 2 The Collaborative Innovation Center of South China Sea Studies, Nanjing University, Nanjing 210093, China; 3 The College of Landscape Architecture, Nanjing Forestry University, Nanjing 210037, China; zj_1229@njfu.edu.cn

**Keywords:** intertidal sand ridge line, radial sand ridges, South Yellow Sea, remote sensing analysis, Dongsha Shoal

## Abstract

The Dongsha Shoal is one of the largest shoals in the South Yellow Sea and has important marine ecological value. The shoal extends in a south–north direction and is controlled by the regional dominating tidal currents. Recently, due to human activities and some natural factors, the geomorphic dynamics of the Dongsha Shoal has undergone drastic changes. However, few people have proposed quantitative research on the changes of tidal flat morphology, let alone the long-term sequence analysis of sand ridge lines. Hence, we attempt to take the Dongsha Shoal in the Radial Sand Ridges as the research area, and analyze the trends of the long-term morphological evolution of the sand ridge lines over the period 1973–2016 based on a high-density time series of medium-resolution satellite images. The sand ridge line generally moves from southeast to northwest, and the position distribution of the sand ridge line from north to south has gradually changed from compact to scattered. We also found that the geomorphological dynamics at different positions of the sand ridge line are inconsistent. The north and south wings are eroded on the west side, while the central area is eroded on the east side. Most of the sand ridge line is moving eastward. In addition, the change of sand ridge line is affected by multiple factors such as sediment supply, typhoon, reclamation and laver cultivation.

## 1. Introduction

A coastal zone is a complex geographic unit of transition between terrestrial and marine ecosystems, and it is also the area responding fastest to global and land-sea changes [1]. Tidal flats, located between the average low tide and average high tide of the coastal zone, are the most important landform system in the coastal zone. They can protect the biodiversity of the coastal zone, resist storm surges, and provide habitat for migrating birds, and provide reserve land resources for coastal economic development [2,3,4,5]. Therefore, the tidal flats have received extensive attention from all over the world [6,7,8,9,10]. Recently, due to global changes and intense human activities, coastal tidal flats have undergone drastic changes, leading to a series of resource and environmental problems. Therefore, it is necessary and urgent to study the dynamic trend of tidal flats and the response to increased human activities and natural factors. The sand ridge line is a watershed divide on the large-scale low tide exposed tidal flat surface. It is the topographic boundary of the adjacent tidal waterway control area, where the rising and falling tides on both sides converge and disperse, and its position and shape change continuously with the regional tidal dynamics, which can reveal the regional dynamic geomorphological process [11].

Along the west coast of the South Yellow Sea from the old Yellow River delta to the Yangtze River estuary in Jiangsu Province, these unique sand ridges have been developing since the late Quaternary [6]. Strong tidal currents, the highest observed tidal range being 9.39 m [12], are the main morphological forces driving the formation of the sand ridges, which cover an area of more than 20,000 square kilometers and are composed of dozens of large sand ridges with corresponding troughs between the ridges [13,14,15]. The tidal ridges are located not too far from the coastline and appear as large areas of silty tidal flats during the ebb tide. This area has huge ecological value for migratory birds given its importance as a coastal habitat.

However, since the Yellow River returned to the Bohai Sea in 1855, the Radial Sand Ridges have lost a large amount of sediment supply [16]. Due to the needs of agriculture, aquaculture, wind power generation and port construction, a large number of coastal beaches in the area have been reclaimed [17]. In addition, global changes and overexploitation of land have caused sea levels to rise, the geomorphological dynamics conditions of the Radial Sand Ridges have changed drastically, and the sedimentation has become erosion [18]. Therefore, an accurate assessment of the evolution of the Radial Sand Ridges is critical to the protection of tidal flat resources and sustainable coastal development. Remote-sensing technology provides nearly continuous monitoring of tidal flats, which are commonly used in many coastal areas around the world [3,4,19,20]. The most representative methods are based on airborne light detection and ranging (LiDAR) and interferometric synthetic aperture radar (InSAR) images [21,22,23,24]. However, due to the high cost of LiDAR-based data and aerial images, it is difficult to perform time series analysis on tidal flats [25,26]. Since the launch of the Landsat-1 satellite in 1972, many satellite images of research value have been archived [27,28]. As more Earth exploration satellites were launched, and other satellite data cooperation projects, the potential of intensive time series analysis has been greatly expanded, especially in tidal flats [29]. Researchers make extensive use of the time series of satellite images, using the waterline detection method (WDM) to construct a digital elevation model (DEM) of the tide plane [30,31]. This method is widely used in many intertidal tidal flats around the world, including the coast of England [32,33,34,35], the Wadden Sea tidal flats, Germany [36,37,38,39], the intertidal zone along the coast of Korea [40,41,42] and China coastal beach [43,44,45,46]. However, few people have proposed quantitative research on the changes of tidal flat morphology, let alone the long-term sequence analysis of sand ridge lines.

In this study, we attempt to take the sand ridge lines of Dongsha Shoal as the research area, analyze the trends of the long-term morphological evolution of the sand ridge lines over the period 1973–2016 based on a high-density time series of medium-resolution satellite images. Our specific goals were (1) to study the long-term evolution of the location of the sand ridge lines, (2) to study the overall trend and change stage of the sand ridge lines (3) to determine the change trend of different positions of the sand ridge lines, and (4) to discuss global changes and the impact of human activities on the landform changes in Dongsha Shoal. This study will help evaluate changes in tidal flats in modern radial sand ridges in Jiangsu Province, provide support for the management of related sea areas, and provide scientific references for sustainable tidal flat development.

## 2. Study Area and Methods

### 2.1. Study Area

**Figure 1:**
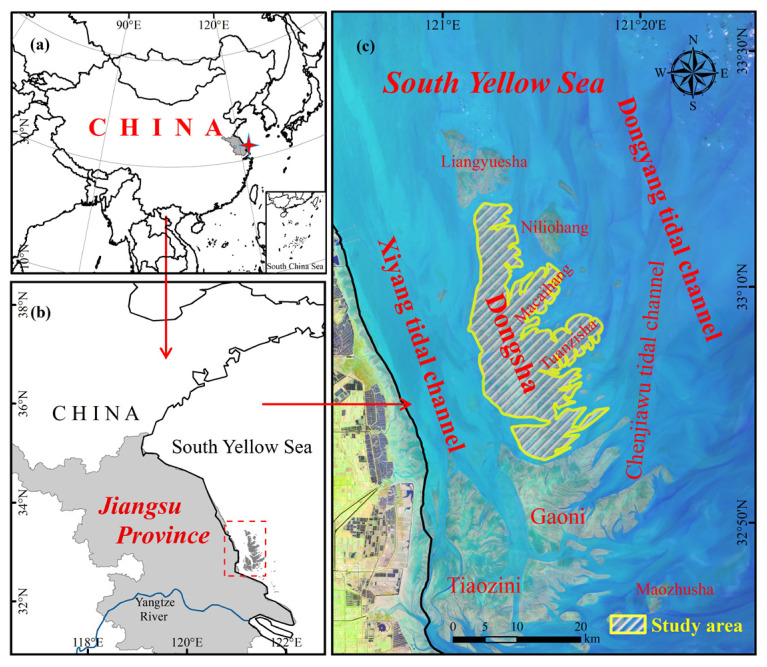
Study area and image of the Dongsha Shoal for a low tide condition. Maps of the Dongsha Shoal (**a**) Mainland China, (**b**) the South Yellow Sea and (**c**) Dongsha Shoal.

Dongsha is the intertidal sandbank with the largest area and the highest mean altitude in this radial sand ridges (Figure 1). The Dongsha Shoal is surrounded by a series of sand ridges and deep trenches. This area has a wide tidal range, broad tidal flats, typical tidal flat landforms, and unstable topography. Tidal flats in the study area are typically more than 25 km in width and have an average slope of approximately 0.18–0.19% [47]. In most periods, the top of the shoal cannot be submerged by tidal flow [48].

The Dongsha Shoal is a macro-tidal area with an average tidal range of 3.9–5.5 m and exhibits an irregular semi-diurnal tide [49]. The distribution of the tidal range has Jianggang at the highest point with a gradual decrease towards the north and south sides [50]. The coastal waters where the shoal is located have the largest tidal range, being more than 3.9 m. The average tidal range of Changsha Port reaches 6.45 m, and the average tidal range of the Dongsha Shoal is 5.44 m. The largest tidal range of 9.39 m was observed on 17 October 2012 at Xintiaoyugang Station [12].

### 2.2. Research Methods

#### 2.2.1. Selection of Remote-Sensing Data and Data Preprocessing

**Figure 2:**
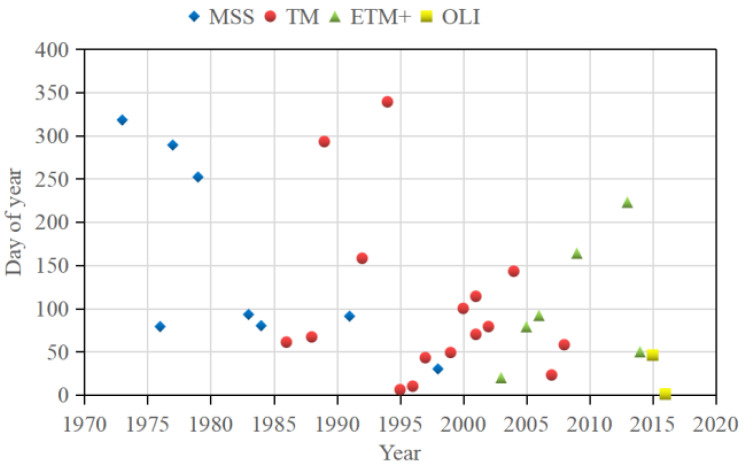
Date of acquisition of satellite imagery used in this study. (Landsat multispectral scanner (MSS); Thematic Mapper (TM); Enhanced Thematic Mapper (ETM+) and Operational Land Imager (OLI)).

The sand ridge line is the watershed divide of the tidal flat exposed on the tidal ridge at low tide, and the return channel water on both sides of the tidal flat enters different tidal channels. The tidal range in the study area is very large, and the low tide exposed tidal flat surface features obvious topographic features. Through the enhanced processing of remote-sensing images and combined with the dynamic characteristics of the tidal current channel on both sides of the tidal flat, the relative position of the tidal channel ends on the tidal flat surface can be visually distinguished, and then the sand ridge line can be extracted. We consulted the tide table, used the US Geological Survey Global Visualization Viewer (GloVis, Reston, VA, USA), and selected the best low-tide middle-resolution remote-sensing images from 1973–2016 through comparison and screening. A total of 33 preview satellite image frames for the research area from 1973 to 2016 were subsequently selected. The specific parameters are shown in Figure 2. These images were acquired by the following sensors: Landsat-1/2/3 Multispectral Scanner System (MSS) (NASA, Washington, DC, USA), Landsat-4/5 Thematic Mapper (TM) (NASA, Washington, DC, USA), Landsat-7 Enhanced Thematic Mapper Plus (ETM+) (NASA, Washington, DC, USA), Landsat-8 (OLI) (NASA, Washington, DC, USA).

The images were pre-processed using ENVI 5.0 (Environmental Systems Research Institute, Redlands, CA, USA) and ArcGIS 10.2 (Environmental Systems Research Institute, Redlands, CA, USA) with both projection transformation and geometric correction (precision of less than one pixel). Meanwhile, image fusion was carried out according to the characteristics of the different sensors. Then interactive stretching in ENVI was used to enhance the images of the study area. The above operations were performed in ArcGIS10.2 to facilitate routine visual interpretation and water body identification, to vectorize the tidal flats and the intertidal sand ridge line, to perform the superimposition analysis and to obtain numerical statistics for the vector elements of the different periods.

#### 2.2.2. Extraction and Analysis Method of Sand Ridge Line

After dealing with the series of 33 images (1973 to 2016) for the Dongsha Shoal, the annual and the accumulated information of the sand ridge line was obtained. In order to study the overall movement trend and movement phase of the sand ridge line, we extracted the midpoint of the sand ridge line and summarized it.

**Figure 3:**
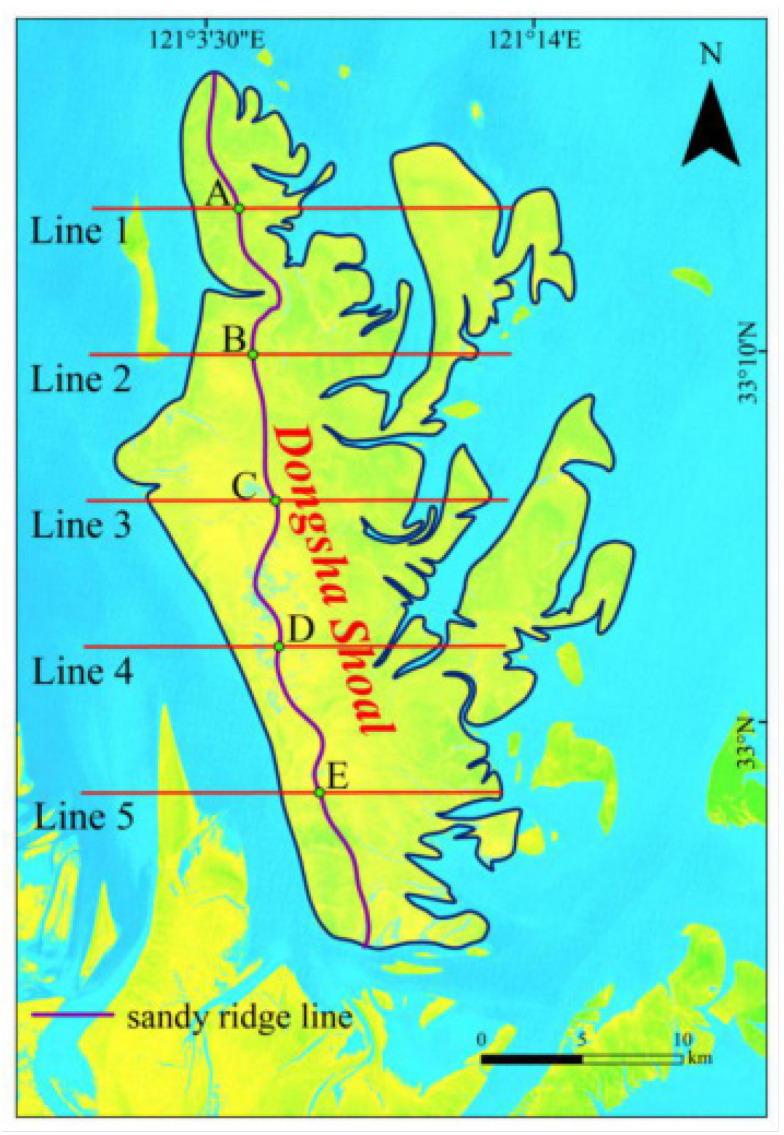
The location of the five typical sections and the sand ridge points formed by their intersection with the sand ridge line.

In order to understand the movement trends of the sand ridge line, we have selected five typical sections, as shown in Figure 3. The positions of the sections are Line 1 (33°13′30″ N), Line 2 (33°9′34″ N), Line 3 (33°5′38″ N), Line 4 (33°1′41″ N) and Line 5 (32°57′45″ N), which traversed the sand ridge line, respectively. Then five sand ridge points (intersection points) A, B, C, D, E and F, were obtained. We counted the locations of sand ridge points, such a yearly sequence for the direction of movement and distance may be summarized and visualized with ArcGIS10.2, thus the spatial distribution characteristics of the intertidal sand ridge lines of the Dongsha Shoal may be assessed.

**Figure 4:**
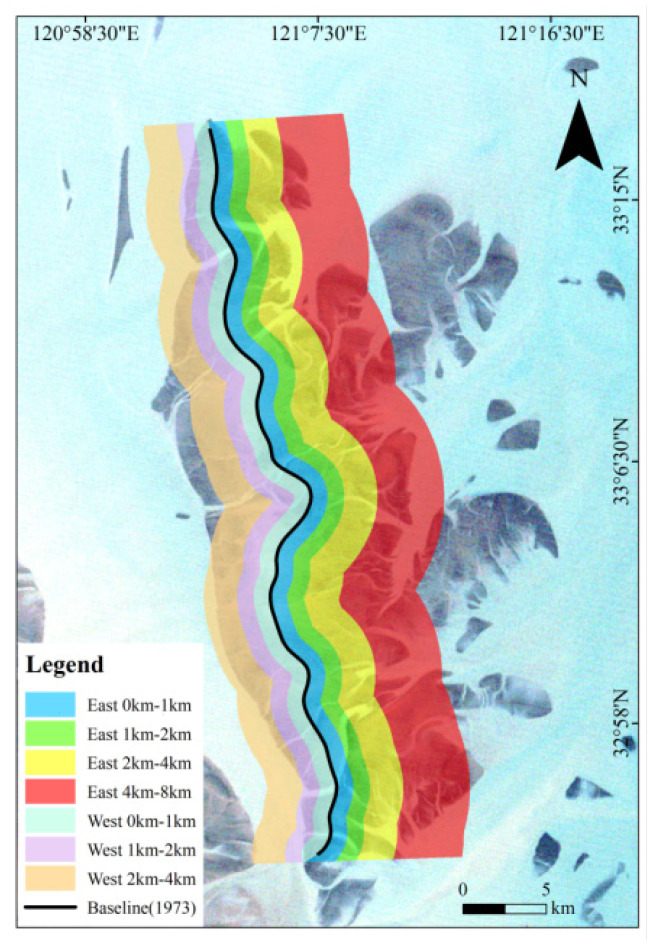
Determination of Dongsha Shoal buffer zone.

In order to study the current distribution and migration trend of the sand ridge line at each stage, we used the buffer analysis and superposition analysis function, taking the sand ridge line in 1973 as the baseline, setting up 1 km, 1–2 km, and 2–4 km buffer zones on the west side, and at the same time setting up 1 km, 1–2 km, 2–4 km, 4–8 km buffer zone on the east side (Figure 4). Finally, the superposition analysis was carried out with all sand ridge lines, and the proportion of the length of sand ridge lines in each buffer zone to the total length is calculated.

Finally, the superposition analysis was carried out with all sand ridge lines, and the proportion of the length of sand ridge lines in each buffer zone to the total length is calculated.

## 3. Results

Dongsha Shoal is a large coastal sandbank, and its degree of evolution is very complicated. In this case, studying the morphological changes of the sand ridge line can deduce the displacement of the Dongsha Shoal, the erosion/deposition caused by the changes in the dynamic conditions of the east and west sides of the Dongsha Shoal, the changes in appearance caused by the combination/separation of Dongsha Shoal and other small sandbanks. Therefore, we used various methods to analyze the evolution trend of the sand ridge line of Dongsha Shoal.

### 3.1. Spatiotemporal Changes of Sand Ridge Lines

**Figure 5:**
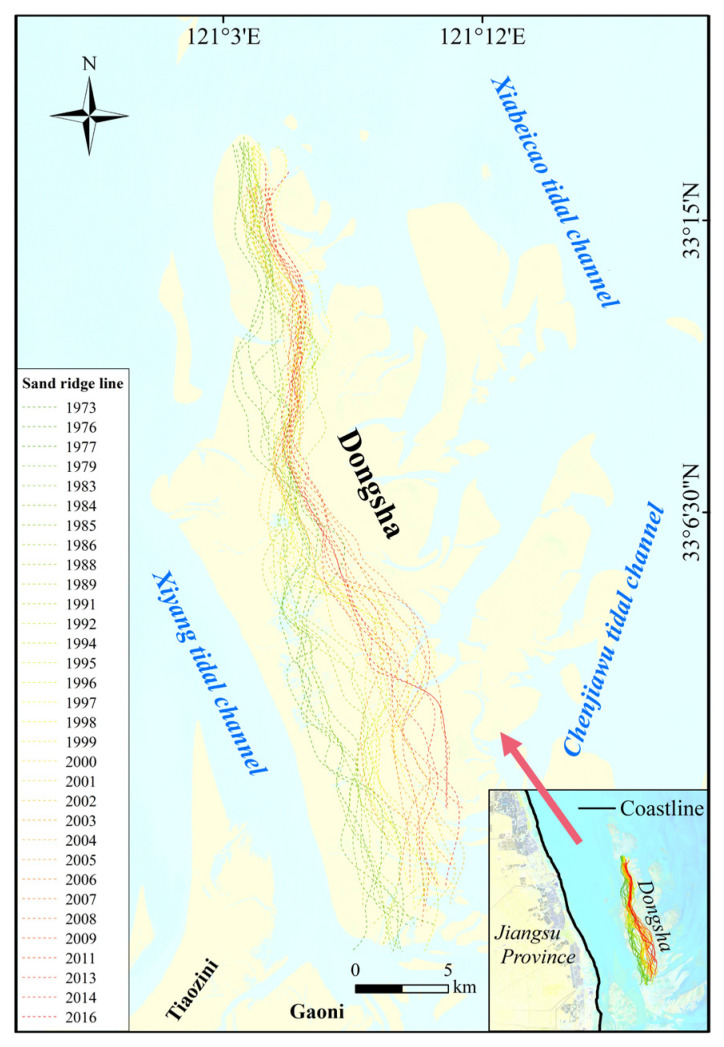
Changes in the sand ridgeline of Dongsha Shoal during 1973–2016.

Dongsha Shoal is a well-preserved core geomorphic unit of the Radial Sand Ridges. The results from Figure 5 show that the sand ridge line of Dongsha Shoal is generally in the north–south direction, basically parallel to the shoreline, and the tidal flats develop unevenly on the east and west sides of the sand ridge line. A large area of tidal flats developed on the east side, with irregular shorelines and generally wide tidal channels. By contrast, the tidal flat area on the west side is smaller and the shoreline is relatively straight.

It can be seen that the strike of the sand ridge line of the Dongsha Shoal is basically stable, indicating that the sedimentary environment and tidal current field in the core area of the Radial Sand Ridges are relatively stable.

**Figure 6:**
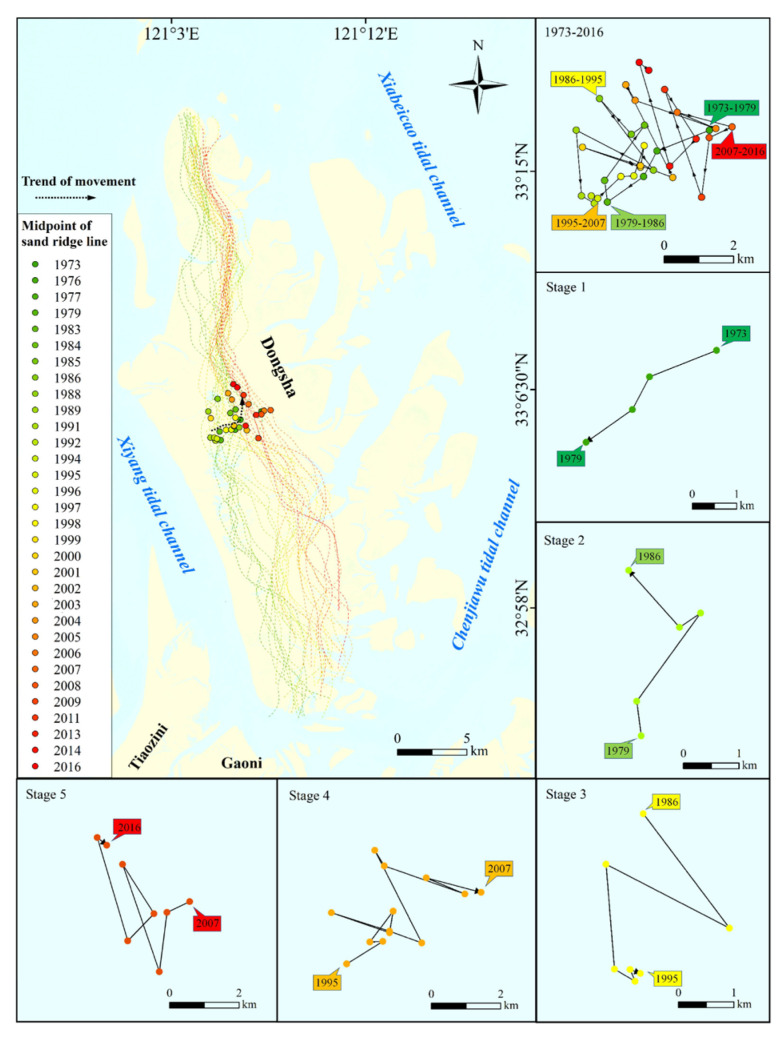
Changes in the midpoint of the sand ridgeline of Dongsha Shoal during 1973–2016.

The midpoint is the typical characteristic point of the sand ridge line. As shown in Figure 6, the change in the position of the midpoint of the sand ridge line clearly expresses the overall movement trend of the sand ridge line. (1) In general, the overall movement trend of the sand ridge line is from southwest to northeast, and the average movement distance is 1.39 km. (2) The sand ridge line does not move in one direction, on the contrary it is constantly oscillating, and with the passage of time, the frequency of the reverse oscillation has a tendency to increase. (3) According to the midpoint movement direction and speed, the sand ridge line movement can be divided into 5 stages. The period from 1973 to 1979 was stage 1, the sand ridge line moved from northeast to southwest with an average speed of 1.25 km/year. It can be seen that there was erosion on the east side of Dongsha Shoal at this time. The period from 1979 to 1986 was stage 2, when the sand ridge line moved from south to north with an average speed of 1.11 km/year. 1986–1995 was stage 3, the sand ridge line moved from north to south, but it began to swing in opposite directions twice, with an average speed of 1.31 km/year. Dongsha Shoal merged with several small sandbanks on the east side, resulting in the direction of movement of the sand ridge line to start to be unstable. The period from 1995 to 2007 was stage 4. The sand ridge line moved from the southwest to the northeast, with 9 reverse swings, with an average speed of 1.39 km/year, indicating that the acceleration of coastal reclamation in Jiangsu Province in the late 1990s changed the marine sedimentary environment around Dongsha Shoal. The erosion of Dongsha Shoal has been intensified while the scarcity of sediment has increased. At the same time, the southern end of the Xiyang tidal channel was also severely eroded. The period from 2007 to 2016 is stage 5. The sand ridge line moves from southeast to northwest, and there are 5 reverse swings, with an average speed of 1.72 km/year. During this period, the Chinese government reclaimed Tiaozini sandbank and Gaoni sandbank, which led to the sediment supply on the south side of Dongsha Shoal being further reduced and the sand ridge line eroding northward.

### 3.2. Variation of Sand Ridge Lines on Typical Section

**Figure 7:**
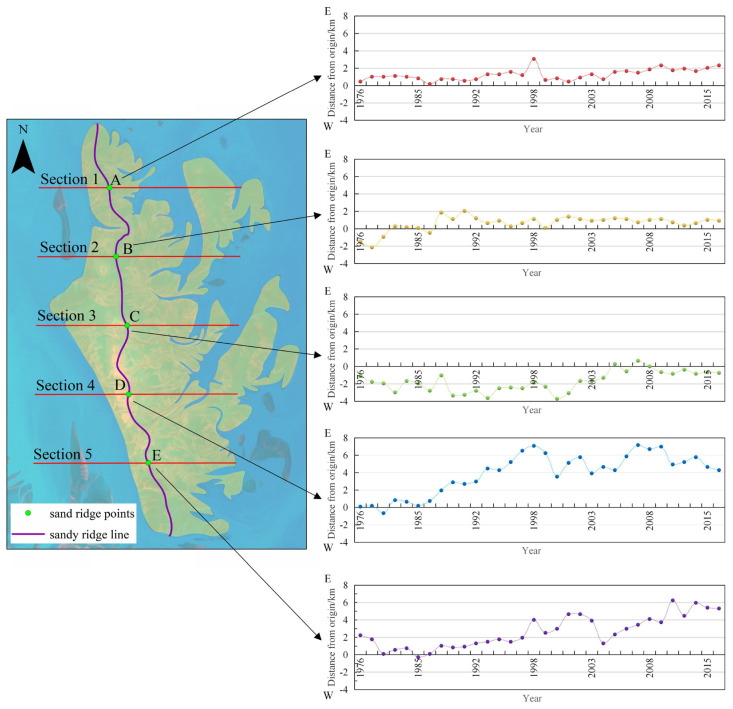
Location distribution of sand ridge points in different periods.

The change characteristics of the different positions of the sand ridge line and the movement speed reflect in detail the scouring and silting situation in the Dongsha Shoal, and reflect the degree and trend of dynamic geomorphology changes in the Dongsha Shoal. The distribution of sand ridge line positions in different periods is shown in Figure 7. The starting position for each point is the point for 1973; this point is located in the quadrant to the east of the origin; the point in the negative quadrant is the point west of the origin.

We use five typical sections to analyze the changes in the spatial position of the sand ridge line.

From the location distribution of the sand ridge points, most of the sand ridge points A, B, D and E are on the east side of the origin, and all points of the sand ridge points A are on the east side of the origin. Most of the sand ridge points C are on the west side of the origin. It can be inferred that the sand ridge line of most periods has a significant westward bulge in the middle section. The farthest position of sand ridge points A on the east side is 3.1 km away from the origin. The farthest position of sand ridge points B on the east side is 2.05 km from the origin, and the farthest position on the west side is 2.14 km from the origin. The farthest position of sand ridge points C on the east side is 0.65 km from the origin, and the farthest position on the west side is 3.72 km from the origin. The movement distance of sand ridge points D and E is obviously longer than sand ridge points A, B and C. The farthest position of sand ridge points D on the east side is 7.17 km from the origin, and the farthest position on the west side is 0.65 km from the origin. The farthest position of sand ridge points E on the east side is 6.25 km from the origin, and the farthest position on the west side is 0.28 km from the origin. Unlike other intersections, most sand ridge points C are located on the west side of the origin. This indicates that the position distribution of the sand ridge line from north to south has gradually changed from compact to scattered.

**Figure 8:**
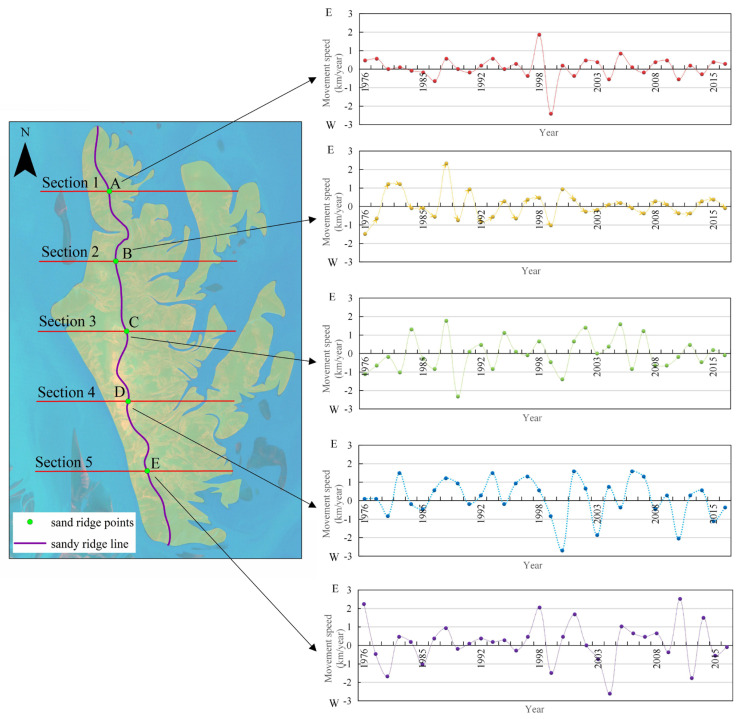
Velocity distribution of sand ridge points movement in different periods.

We analyze the movement speed of the sand ridge points. As shown in Figure 8, the average speed of the sand ridge point of Dongsha Beach is 0.04 km/year (to the east), and the maximum speed of the sand ridge point is 2.70 km/year (to the east). Among them, the fastest part of the sand ridge point is in the south (Section 5), with an average speed of 1.66 km/year (to the east). The slowest part of the sand ridge point is in the middle (Section 3), with an average speed of 0.23 km/year (to the west).

**Figure 9:**
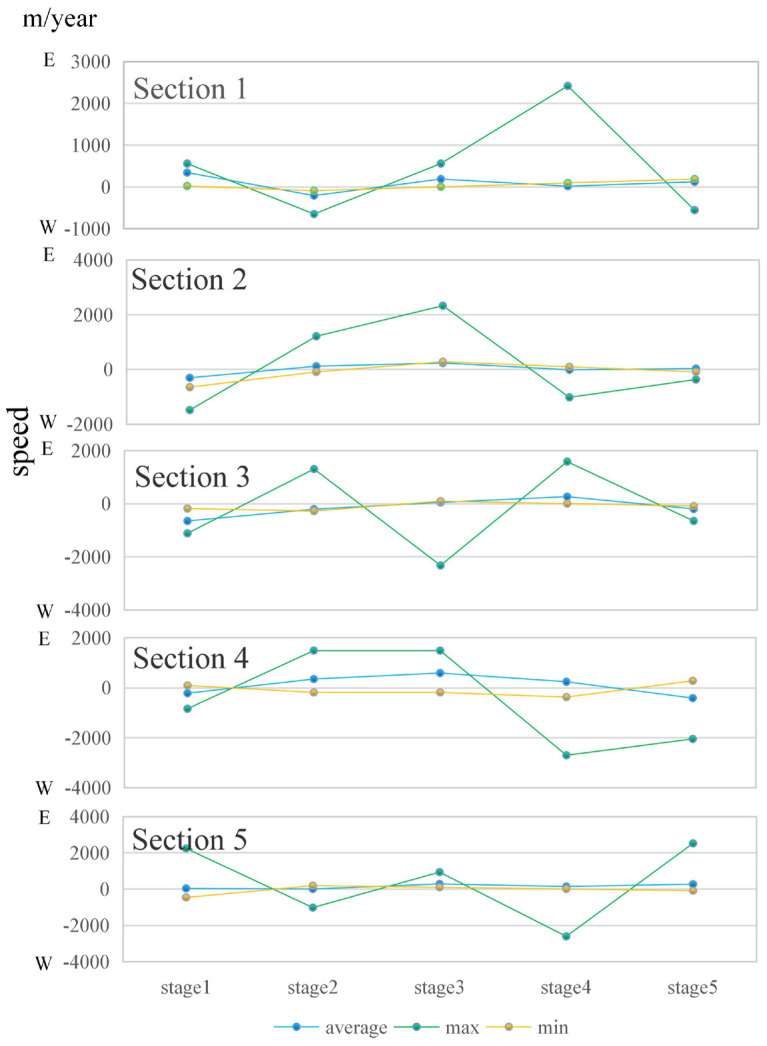
Movement of sand ridge line in typical sections.

As shown in Figure 9, since the velocity and direction of the sand ridge points on each section are constantly changing, the overall speed of the movement is represented as a low-speed eastward movement. The movement direction of the sand ridge point A presents the law of east–west–east–west–east, and the degree of velocity fluctuation gradually increase. The direction of movement of the sand ridge point B presents a law of west-east-east-west-east, and the amplitude of velocity fluctuations is first large and then small. The movement direction of the sand ridge point C presents the law of west–west–east–east–west, and the speed fluctuation range is firstly large and then small. The movement direction of the sand ridge point C presents the law of west–east–east–east–west, and the velocity fluctuations are first small and then large. The direction of sand ridge point C movement on Section 3 presents the law of west–west–east–east–west, and the degree of velocity fluctuation is first large and then small. The movement direction of the sand ridge point D presents the law of west–east–east–east–west, and the velocity fluctuations are first small and then large. The movement direction of the sand ridge point E presents a regular eastward, and the speed and direction are relatively stable. It can be seen that the fluctuation degree of the speed of the sand ridge line gradually decreases from north to south.

### 3.3. Analysis of Sand Ridge Lines Migration Trend

**Figure 10:**
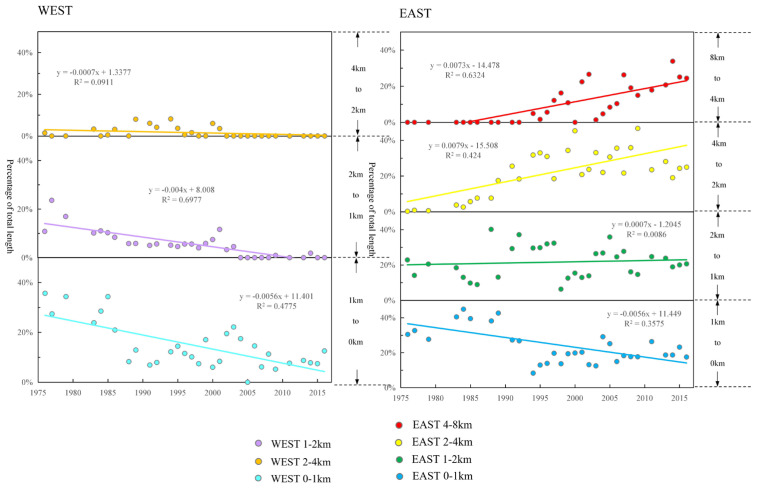
Overlay analysis of sand ridgelines buffer zone.

In order to study the migration trend of the sand ridge line in the east–west direction, we took the sand ridge line in 1973 as the baseline, and calculated the proportion of the length of the sand ridge line in each buffer zone at each time phase to the total length, summarized the data to obtain Figure 10. The results are as follows: (1) Since 1976, the west side of Dongsha Shoal has been continuously scoured. In this case, the sand ridge line has been moving eastward, and there is a tendency to move within the area 2–8 km east of the baseline. (2) In most time phases, the proportion of the length of the sand ridge line on the east side of the baseline exceeds 50% and gradually increases. (3) The hydrodynamic force of the Xiyang Tidal Channel continues to increase. The east bank of Dongsha Shoal was gradually eroded, causing the sand ridge line to move eastward. After 2004, most of the sand ridge line has moved 1 km to 4 km west.

## 4. Discussion

### 4.1. Influence of Natural Factors on the Change of Sand Ridge Line in Dongsha Shoal

Several natural factors have obvious influence on the evolution of the sand ridge line.

#### 4.1.1. Changes in Sediment Load

In 1128, the Yellow River captured the Huai River from the northern part of Jiangsu Province into the sea. A large amount of sediment caused the coasts of most parts of Jiangsu to move rapidly toward the sea [51,52]. Since the Yellow River moved to the Bohai Sea due to natural destruction in 1855, the source of sediment from the Yellow River into the South Yellow Sea has been greatly reduced, and the hydrodynamic conditions of the Dongsha Shoal have changed [53,54]. As a result, the radial sand ridge has entered an unprecedented adjustment stage, causing severe ocean erosion and coastline retreat on the central coast of Jiangsu and the west side of the Dongsha Shoal [55,56]. This is also the driving force for the overall eastward migration of the sand ridge line.

#### 4.1.2. Changes in Sedimentary Dynamics

Since 1855, the sedimentary dynamics of the radial sand ridge area has changed greatly. The impact of runoff and sediment load on tidal flats gradually weakened, and the leading role of offshore tidal currents gradually increased. The Xiyang tidal channel is a coastal tidal channel in the northern flank of the radiating sandbar, extending to the south, causing the low tidal flat to be eroded under the influence of nearshore hydrodynamic forces. Under the action of the coastal currents in Jiangsu Province, the erosion material is transported southward [6]. In addition, the inner area of the radial sand ridge is present on the strong tidal current coast. When the strong tidal current hits the concave bank of the tidal flat, this will cause the erosion of the tidal flat and the repeated swing of the sand ridge line.

Global warming will accelerate sea level rise and increase the intensity and frequency of storm surges and waves, which will lead to the possibility of increased coastal erosion. Historical records of tides indicate that the average rate of sea level rise along the the Radial Sand Ridge is about 5.26 mm/year, which is about twice the global average (2.43 mm/year) [57]. In the next 30 years, the average sea level along the Radial Sand Ridge will rise by about 85–150 mm [28]. This rise will lead to a decrease in the area of the tidal flat exposed at the lowest tide in the Dongsha Shoal, the expansion of the erosion area, and the ridge line of the sand will become unstable.

#### 4.1.3. Impacts of Typhoon

Tropical cyclones are an important driving factor for short-term catastrophes in the Dongsha Shoal [58]. The typhoon information was derived from the Tropical Cyclone Best Track Dataset issued by the Shanghai Typhoon Institute of the China Meteorological Administration [59].

**Figure 11:**
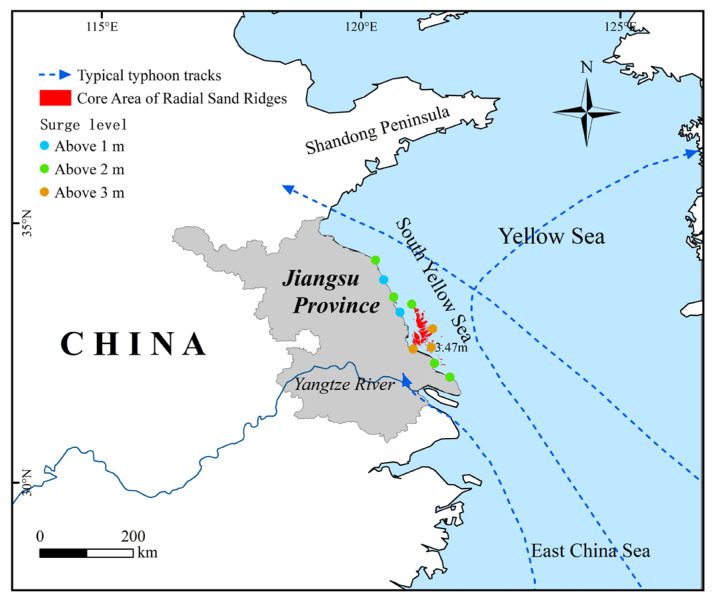
Typical typhoon tracks along the Jiangsu coast.

After summarizing, we found that there are mainly three typical paths of typhoons affecting the coastal areas of Jiangsu. The typical paths are shown in Figure 11. The storm surge levels caused by typhoons making straight landfall, by typhoons active in the offshore area, and by typhoons moving northward after landfall were high. In terms of spatial distribution, the radial sand ridge area was seriously affected by the three types of typhoon and the storm surge was the largest, with the highest water level of 3.47 m. Studies have shown that before and after the passage of a typhoon, the shape of the tidal flat changes greatly, mainly manifested by the sudden change of the tidal flat area and the appearance of new tidal channel [60].

**Figure 12:**
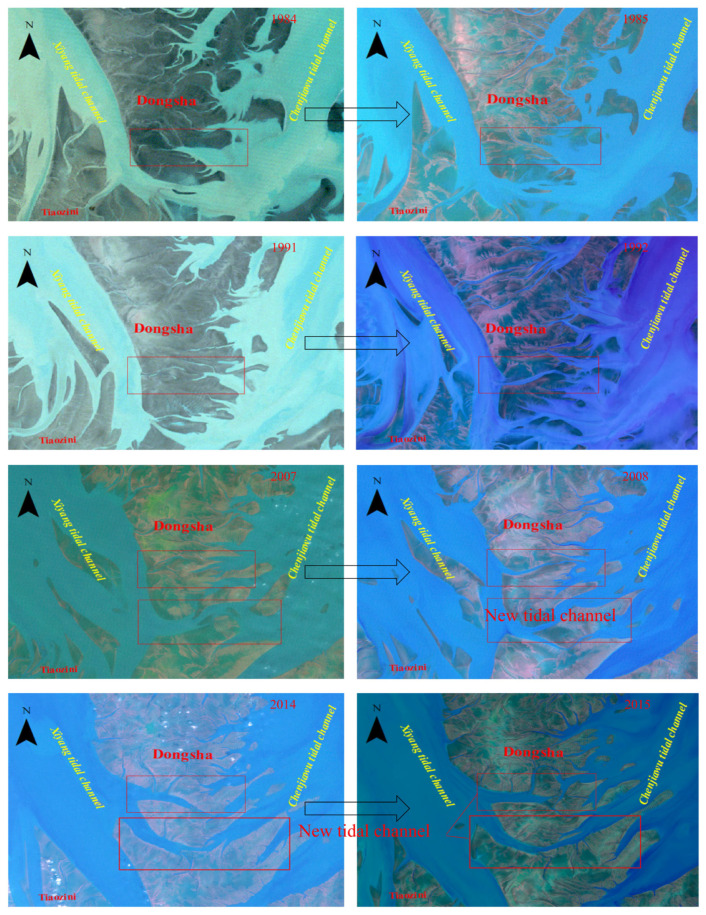
Several cases show the response of the Shaling Line to the typhoon.

As shown in Figure 12, several typical examples show the drastic changes on the south side of the Dongsha Shoal after the typhoon passed. The red box marks the position of drastic changes. Among them, the large tidal trench in the south of the Dongsha Shoal in 1985 was wider than in 1984. In 1992, a tidal trough that run through the east and west banks appeared for the first time in the south of the Dongsha Shoal under the impact of typhoon. In 2008, under the influence of the transit typhoon, two tidal troughs connecting the east bank and the west bank were formed, and the tidal trough on the south side gradually developed into a tidal channel. In 2015, the tidal trough on the north side gradually developed into a tidal trough. The shoal between the two tidal troughs was separated from the Dongsha Shoal, causing the south bank of the Dongsha shoal to move northward, and the southern end of the sandy ridge line also moved north.

### 4.2. The Impact of Human Activities

#### 4.2.1. Port Construction

Port construction along the Jiangsu coast has also accelerated since the 1980s and stimulated the reclamation process of the tidal flats. Taking the Yancheng coastal wetland as an example, in the past decade the ports of Binhai, Sheyang and Dafeng have expanded. the changes of water dynamic environment due to the massive coastal engineering contributes to the worsening erosion at the western tidal flat of Dongsha Shoal [61].

#### 4.2.2. Reclamation Activities

Tidal flats are an important part of coastal wetlands in Jiangsu Province and one of the most important natural resources. In order to alleviate the situation of large population and small land in Jiangsu Province, coastal areas have been carrying out long-term tidal flat reclamation projects. Historically, the reclamation area along beaches in Jiangsu Province has reached 2 × 10^6^ hm^2^ [62]. Since 1949, large-scale reclamation in Jiangsu has not only occupied a large amount of tidal flat resources, but also changed the offshore sedimentary environment [6].

In recent reclamation projects, a number of long dykes has cut off the connection between the inner tidal flats and the outer seawater. The dyke construction has changed radically not only the estuarine tidal system inside the dyke, but also the coastal marine environment outside the dyke (Figure 12).

**Figure 13:**
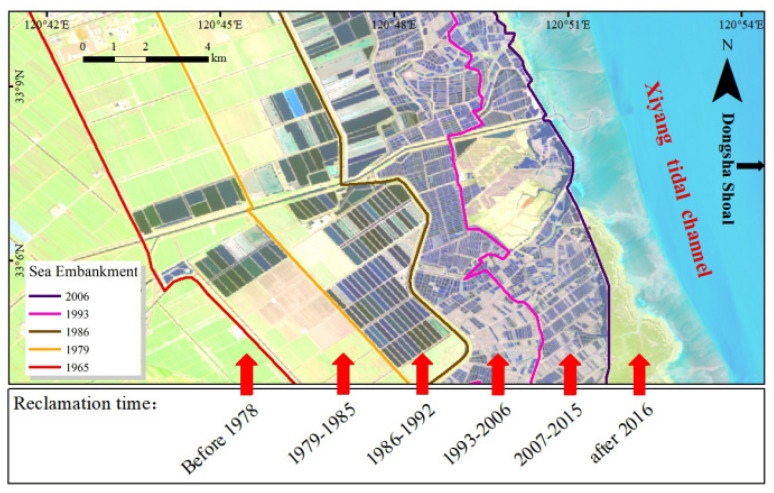
The history and distribution of tidal flat reclamation in Jiangsu Province.

When a seawall is built, the rich supply of sediment will provide more sediment to the new beach, so that the beach gradually becomes silted and then gradually extends outward (Figure 13). The result is that the construction of the seawall narrows the western tidal cross section, under the premise of constant tidal flux, tidal range will increase. After the completion of the first and second stage’s reclamations, the peak velocity and unit tidal discharge in Xiyang tidal inlet increased about 20% and 5~10% [63].

**Figure 14:**
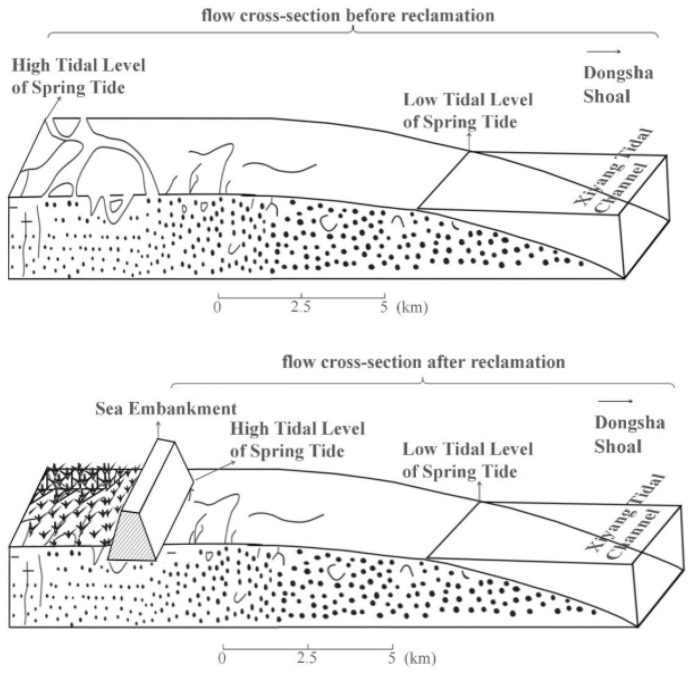
Representation of the water flow cross sections of the Xiyang tidal channel before and after reclamation.

As shown in Figure 14, when the flow rate increases at the same time, the flow rate in the waterway will inevitably become larger, resulting in erosion by the watercourse on both sides of the channel such that the water channel is widened. In this respect the Dongsha Shoal is a typical example. A large amount of sediment in the embankment before the seawall gradually becomes silted hence the sediment content of the western seawater gradually decreases [14]. Thus, the increase in the tidal current and the decrease in the sediment content in seawater have led to an increase in the erosion of the west bank of the Dongsha Shoal.

**Figure 15:**
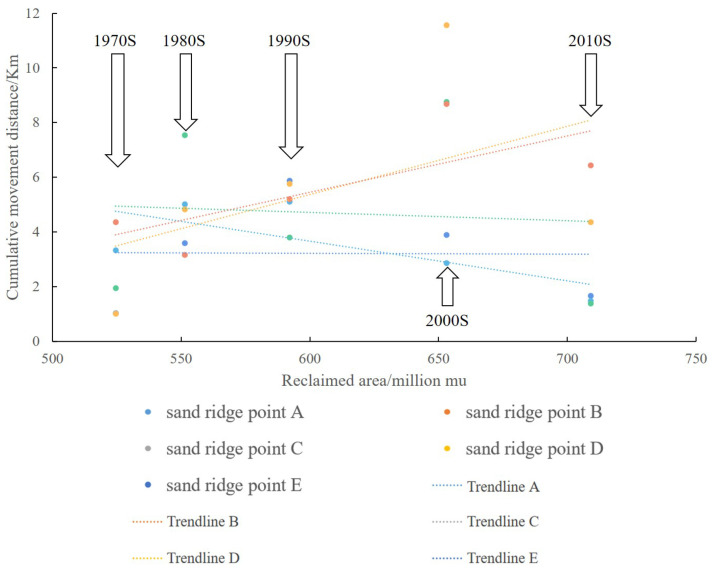
The response of sand ridge points movement of Dongsha Shoal to the reclamation in Yancheng.

In order to quantitatively analyze the response of sand ridge points movement of Dongsha Shoal to the reclamation, we searched for the data of Jiangsu coastline reclamation since modern times, and calculated the cumulative reclamation of the Yancheng coast in the 1970s, 1980s, 1990s, 2000s and 2010s area [64]. Then the relationship between sand ridge point movement and reclamation was analyzed, and the result is shown in Figure 15. The beach reclamation activities on the coast of Yancheng have a greater impact on the movement of the sand ridge line. According to the linear relationship between cumulative movement distance and cumulative reclamation area of sand ridge points in different periods, the influence process can be expressed as follows: With the increase of tidal flat reclamation area, the cumulative movement distances of sand ridge points A, D, and E show an increasing trend. By contrast, the cumulative moving distance of sand ridge points B and C shows a decreasing trend. It can be seen that the tidal flats speed up the movement of the northern and southern parts of the sand ridge line, while the movement speed of the central part slows down.

#### 4.2.3. Laver Cultivation

Human activities such as the rapid development of marine aquaculture in the central Jiangsu coast have had a marked impact on the tidal flat morphology. The Dongsha Shoal has unique resource advantages such as good light conditions and no pollution, and has excellent conditions for breeding *Porphyra yezoensis*.

**Figure 16:**
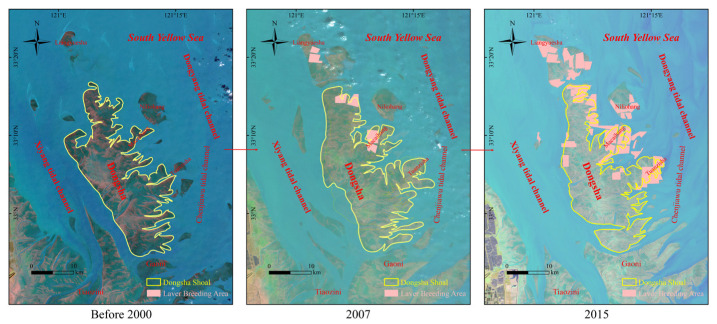
Representation of the water flow cross sections of the Xiyang tidal channel before and after reclamation.

As shown in Figure 16, existing studies have shown that *Porphyra yezoensis* breeding areas began to appear in the Dongsha Shoal at the beginning of this century. In 2007, the area was 75.82 hectares, and in 2015 it further increased to 954.08 hectares [65]. The east side of the Dongsha Shoal faces the open sea. Under the action of northeast wave and east wave, the sediment moves west and southwest with the high tide water, and accumulates on the beach surface, making the east side of the Dongsha Shoal continue to grow. In this century, humans began to cultivate laver at the low-tide beach in the northeastern part of the Dongsha Shoal. The large area of laver cultivation nets has slowed down the northeast and eastward waves to a certain extent and blocked part of the sediment from going to the middle and high tide beaches. The advancement of the shoal slowed down the siltation speed of the high tide beach and the middle tide beach, and the sand ridge line also appeared to move to the northeast as a whole.

### 4.3. Ecological Value and Protection Strategy of the Dongsha Shoal

The Dongsha Shoal has great value in the support of wetland function and biodiversity. With reference to coastal development, the government has given much attention to the promotion of coastal development and the ensuing economic benefits but, at the same time, has neglected the need to protect the Dongsha Shoal. The development activities in the Yancheng coastal area have caused some disturbance to the intertidal natural environment. Reclamation projects can cause a sizeable and adverse threat to the ecology of a region, and the government has now realized that land reclamation must be supported by a rigorous environmental impact assessment and sound scientific and technological support. The Migratory Bird Sanctuaries along the Coast of the Yellow Sea–Bohai Gulf of China (Phase I) was selected as as part of the World Heritage List in 2019 and the Radial Sand Ridges is the core protection area of this heritage site, which means that the protection of the Dongsha Shoal will receive more support from the government. For now, the construction of artificial islands and land reclamation has basically stopped and protection measures for the Dongsha Shoal wetlands are in place and overseen by the government.

## 5. Conclusions

Scientific analysis and demonstration of the evolution and the factors that influence the sand ridge line of the Dongsha Shoal will aid in understanding the changes in the tidal flat in modern radial sand ridges in Jiangsu Province and achieve sustainable use of the tidal flat.

In this study, based on a novel remote-sensing method, we explored the detailed temporal and spatial dynamics of the modern Dongsha Shoal landform evolution through the location of sand ridge lines obtained from 33 scenes of Landsat satellite images of the study area from 1973 to 2016. Our results show that in the past 33 years, the strike of the sand ridge Line has been basically stable, and the overall movement trend of the sand ridge line is from the southwest to the northeast. From the change of the sand ridge line it can be inferred that the dynamics of the geomorphology are inconsistent. The north and south wings are eroded on the west side, while the central area is eroded on the east side. Most of the sand ridge line is moving eastward. In addition, the change of sand ridge line is affected by multiple factors such as sediment supply, hydrodynamics and reclamation.

This research has been based on medium-resolution remote-sensing satellite image analysis and the discussion has focused on the dynamic changes of the sand ridge line of the Dongsha Shoal. Limitations of the study relate to the satellite repeat cycle and the level of cloud cover; yet to solve some problems, such as the monitoring of the sand ridge line of the Dongsha Shoal, seasonal changes for the region, which are currently lacking, are needed. Due to the limitations of this research, this paper does not draw clear conclusions on these issues. In the future, higher-resolution satellite images will be used to conduct in-depth research on the above issues in combination with a seasonal biodiversity survey so that countermeasures and suggestions for in situ conservation can be put forward, in order to achieve sustainable development of the tidal flat of Jiangsu Province.

## Data Availability

Publicly available datasets were analyzed in this study. This data can be found here: http://glovis.usgs.gov/, http://tcdata.typhoon.org.cn/zjljsjj_zlhq.html.
